# Ordered Mesoporous Silica Prepared with Biodegradable Gemini Surfactants as Templates for Environmental Applications

**DOI:** 10.3390/ma18040773

**Published:** 2025-02-10

**Authors:** Sarvarjon Kurbonov, Martin Pisárčik, Miloš Lukáč, Zsolt Czigány, Zoltán Kovács, István Tolnai, Manfred Kriechbaum, Vasyl Ryukhtin, Viktor Petrenko, Mikhail V. Avdeev, Qiang Tian, Ana-Maria Lacrămă, László Almásy

**Affiliations:** 1 HUN-REN Centre for Energy Research, Konkoly-Thege Miklós út 29-33, 1121 Budapest, Hungary; sarvarjon@student.elte.hu (S.K.); czigany.zsolt@ek.hun-ren.hu (Z.C.);; 2 Department of Chemical Theory of Drugs, Faculty of Pharmacy, Comenius University, SK-83232 Bratislava, Slovakia; pisarcik@fpharm.uniba.sk (M.P.);; 3 Institute of Inorganic Chemistry, Graz University of Technology, 8010 Graz, Austria; manfred.kriechbaum@tugraz.at; 4 Nuclear Physics Institute, Czech Academy of Sciences, 250 68 Husinec-Řež, Czech Republic; ryukhtin@ujf.cas.cz; 5 BCMaterials—Basque Center for Materials, Applications and Nanostructures, UPV/EHU Science Park, 48940 Leioa, Spain; viktor.petrenko@bcmaterials.net; 6 IKERBASQUE—Basque Foundation for Science, 48011 Bilbao, Spain; 7 Frank Laboratory of Neutron Physics, Joint Institute for Nuclear Research, Joliot-Curie 6, 141980 Dubna, Russia; avd@nf.jinr.ru; 8 State Key Laboratory of Environment-Friendly Energy Materials, Southwest University of Science and Technology, Mianyang 621010, China; 9 “Coriolan Dragulescu” Institute of Chemistry, Bv. Mihai Viteazul, No. 24, 300223 Timisoara, Romania

**Keywords:** sol–gel, nanoparticle, MCM-41, SANS, SAXS, TEM, Pb(II), silica, mesoporous, gemini surfactant

## Abstract

Mesoporous silica sieves have been prepared through sol–gel synthesis using diester gemini surfactants as pore templates, aiming to obtain new materials with potential use for water remediation. A series of mesoporous spherical silica particles of submicron size have been prepared in an alkali-catalyzed reaction, using a tetraethyl orthosilicate precursor and bis-quaternary ammonium gemini surfactants with diester spacers of varied lengths as pore-forming agents. The effect of the spacer length on the particle morphology was studied using nitrogen porosimetry, small-angle X-ray scattering (SAXS), ultra-small-angle neutron scattering, scanning, and transmission electron microscopy (SEM, TEM). The results revealed that for all spacer lengths, a long-range hexagonal pore ordering developed in the materials. The silica particles were nearly spherical, with sizes below 1 micrometer, and a weak dependence of the mean particle size on the spacer length could be observed. The template removal procedure had a strong influence on the porosity: calcination caused a moderate shrinkage of the pores while retaining the hexagonal structure, whereas treatment with acidified ethanol resulted in only partial removal of the surfactants; however, the hexagonal structure was severely destroyed. The applicability of the obtained calcined materials as adsorbents for heavy metal ions from water was studied with the example of Pb(II). A high sorption capacity of 110 mg/g was obtained in batch experiments, at pH 5 and 4 h contact time.

## 1. Introduction

The development of novel porous materials has been strongly encouraged due to their wide range of applications in chemical technology, e.g., in the fields of adsorption, separation, and catalysis applications. The design, synthesis, and modification of porous materials are in some respects more challenging than the synthesis of dense materials. Therefore, new strategies and techniques are continuously being developed for the synthesis and structure tailoring of mesoporous materials [1,2,3].

Mesoporous materials based on silicates or aluminosilicates with ordered porosity are commonly synthesized using liquid crystal mesophases, formed by ionic or polymeric surfactants [4]. The first surfactant used in the fabrication of MCM-41 (Mobil Composition of Matter No. 41) in the early nineties was hexadecyltrimethylammonium chloride [5]. In the subsequent research, hexadecyl trimethylammonium bromide (CTAB) became the favorite standard pore-forming surfactant for this type of material. In the same decade, Voort et al. proposed the use of more complex templating molecules: ionic gemini surfactants [6]. Gemini or dimeric surfactants are composed of two hydrophilic heads, two hydrophobic tails, and a spacer interconnecting the hydrophobic moieties [7]. Synthesis conditions in general, and the molecular structure of the pore template molecules in particular, are important for determining the properties of prepared mesoporous materials [8,9]. Following the success in the preparation and application of CTAB-based mesoporous materials, gradually, biocompatibility and sustainability factors have also contributed to the development of many new types of surfactants containing eco-friendly biodegradable functional moieties [10]. To this end, gemini surfactants with biodegradable urea, carboxamide, or ester groups are particularly interesting as templating agents in the preparation of ordered mesoporous silica.

Many studies explored the capability of various gemini surfactants to tailor the pore structure of ordered mesoporous silica. Using gemini surfactants with polymethylene spacers (abbreviated usually as C_n-s-m_ or simply n-s-m with the general formula [C_n_H_2n+1_N^+^(CH_3_)_2_–(CH_2_)_s_–N^+^(CH_3_)_2_C_m_H_2m+1_] 2Br^−^), Voort et al. [6] obtained pure mesoporous silica of MCM-48 and MCM-41 types. As in the case of monomeric surfactants, the alkyl chain length (*n*, m) determines the pore size, whereas the length of the spacer (s) influences the micelle morphology and, hence, the resulting crystallographic phase of the porous silica. Specifically, a spacer length of 10−12 units yielded a cubic MCM-48 structure, whereas shorter spacers are more conducive to the formation of the hexagonal MCM-41 structure. Both 18-12-18 and 16-12-16 gemini surfactants yielded MCM-48, with a surface area in the range of 1200−1600 m^2^/g and pore volumes exceeding 1.2 mL/g; the 18-10-18 and 16-10-16 surfactants also yielded MCM-48, albeit the quality of these materials was lower, and the 16-8-16 surfactant resulted in an MCM-41structure [6].

In another study, Yang et al. used two symmetric gemini surfactants with short polymethylene spacers, (1,3-bis(hexadecyldimethylammonio)-propane dibromide, (1,3-bis(3-hexadecylimidazolium-1-yl) propane dibromide, and a self-designed asymmetric compound (1-(3-(hexadecyldimethylammonio)prop-1-yl)-3-hexadecylimidazolium dibromide) as template agents. They obtained high surface area MCM-41 materials with uniform hexagonal pore structure [11]. Moreover, the silica obtained using the self-designed asymmetric gemini surfactant possessed the largest surface area (880 m^2^/g) and was further used as a sorbent for the organic dye crystal violet, showing a remarkable sorption capacity of over 450 mg/mL [11].

Hu et al. [12] prepared titanium-doped mesoporous silica using the symmetric C_16-6-16_ surfactant as a template in alkaline conditions, varying the Ti/Si molar ratio and obtaining a variety of morphologies. At low Ti content, a well-formed MCM-41 structure was developed, while high Ti/Si molar ratios prohibited the formation of an ordered pore structure [12].

A series of mesoporous silica spheres with radially oriented pore structures and tunable particle sizes were synthesized by the self-assembly of cationic gemini surfactants and silica precursors under basic conditions. By varying the initial synthesis conditions, such as molar composition, temperature, and addition of a co-solvent, the particle sizes were controlled in a range of 120–490 nm, while the pore sizes changed from 2.2 to 3.4 nm. With an increase in the concentration of ethanol, the sphere diameter varied from 70 to 460 nm, and the increase in reaction temperature from 0 to 90 °C led to the average diameter of the silica spheres to change from 90 to 410 nm. Higher NaOH concentration and lower surfactant concentration facilitated the formation of larger particle sizes [13]. A particle diameter of 180–380 nm was achieved with the addition of co-solvents, such as isopropanol, acetone, acetonitrile, dimethyl sulfoxide, and methanol [13].

Ordered porous silicas with pores in the micro–mesoporous range can also be synthesized by using assemblies of gemini surfactants as pore templates. Depending on the surfactant type (e.g., symmetric or asymmetric) and reaction conditions, a variety of phases can be obtained. In the study of Romero et al. [14], only hexagonal phases developed in acidic conditions, while under basic conditions, surfactant 12-2-12 produced a cubic symmetry MCM-48 material, whereas the asymmetric surfactant 8-2-16 yielded hexagonal MCM-41, both of high quality, as evidenced by four strong low-angle X-ray diffraction peaks [14]. These results also illustrate that not only the overall hydrocarbon content but also the dissymmetry of the surfactants determines their liquid crystalline mesophases. Moreover, in the presence of a silica source, inorganic structures of various morphologies can be obtained [14].

Further applications of gemini surfactants for various types of silica were explored by Zhang and coworkers [15,16], who prepared hollow mesoporous silica nanoparticles with pore sizes in the range of 2.2–3.7 nm and specific surface areas up to 1360 m^2^/g using both symmetric and asymmetric C_n-2-m_ surfactants. Prominently, no co-solvent was needed in the synthesis procedure; only tetraethoxysilane as a silica source, along with the surfactant, water, and NaOH as the catalyst. The developed method provided nanoparticles with easily controllable pore sizes for versatile applications in catalysis and drug delivery [15,16].

The majority of previous research focused on the synthesis of ordered mesoporous materials using gemini surfactants with different alkyl tails and testing different kinds of spacer chains. In this present work, we applied a symmetric gemini surfactant with a biodegradable diester spacer for the synthesis of ordered mesoporous silica. We focused on the study of controlled changes in the morphology of the porous silica by varying a single parameter, the length of the spacer chain, within a broad range from 2 to 8 methylene groups. This way, the diameters of the elongated pores could be kept unchanged, whereas the changes in the spacer affected the micelle formation of the surfactant, and subsequently the morphology of the porous silica. The synthesis has been performed in an alkaline catalyzed reaction at ambient conditions. Cationic bisammonium gemini surfactants, with two dodecyl chains and a diester spacer, were selected as pore-forming templates. The structure of the resulting materials was determined from subnanometer to micrometer dimensions using nitrogen porosimetry, small-angle X-ray scattering, ultra-small angle neutron scattering, and electron microscopy. Further, their adsorption performance has been evaluated toward selected metal ions from aqueous solutions and promising results were obtained for further use of the developed materials for environmental applications.

## 2. Experimental

### 2.1. Synthesis

**Figure 1:**
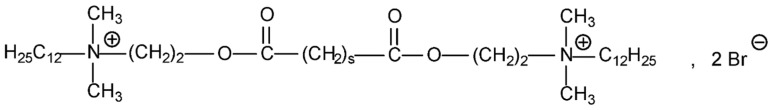
Molecular structure of the gemini surfactants with diester spacer.

Tetraethoxysilane (TEOS, 99%, for analysis, Merck & Co., Inc., Rahway, NJ, USA), ethanol (99%, SC Mobil Dacami SRL, Simeria, Romania), ammonia solution 25% (Silal Trading SRL, Bucharest, Romania), hydrochloric acid ~37% (Sigma-Aldrich, Schnelldorf, Germany), and diester gemini surfactants were used for the synthesis of the mesoporous silica. The diester gemini surfactants C_12-s-12_ were synthesized by the reaction of alkane-diylbis (bromoethylacetate) with dimethyl-dodecylamine. Their molecular structure is shown in Figure 1. More details are given in the original publication of Pisárčik et al. [17].

Six mesoporous silica samples were synthesized by the modified Stöber method [18], using gemini surfactants of varying spacer lengths as pore templates, TEOS in ethanol and water mixture, and base catalyst NH_3_. In a typical synthesis, 0.3 g of surfactant was added to 20 mL of distilled H_2_O and 5.0 mL of ethanol. The mixtures were tempered in an oven at 35 °C for 15 min, then taken out and stirred for 30 min at room temperature. Then, 3 mL of aqueous ammonia solution (25%) was added to the mixtures, and stirring continued further for 5–10 min at room temperature. After that, 1.5 mL of the silica precursor TEOS was poured into the solution dropwise using a plastic transfer pipette under magnetic stirring. Stirring continued for 3 h at room temperature.

After one day, the solid product was recovered by filtration and washed several times with distilled water with repeated filtrations until the pH of the washing water approached the pH value of the distilled water. For each sample, appr. 3 L/g of water was used. Next, all the samples were dried at room temperature and subsequently dried at 100 °C. These samples were labeled as S-e“x”-100, where “x” is the number of methylene groups in the spacer of the gemini molecule.

The surfactant template was removed from the silica in two ways: calcination at 540 °C for 5 h (1 °C/min heating rate), and solvent extraction, and the samples were named S-e“x”-540 and S-e“x”-e, respectively. For solvent extraction, 0.15 g of xerogel was treated with a mixture of 20 mL ethanol and 1 mL HCl 0.1 M, with stirring for 3 h at 50 °C. Next, the materials were filtered, washed with 50 mL of ethanol, and dried at room temperature. The next day they were put for drying at 100 °C for 24 h.

### 2.2. Characterization

FT-IR (Fourier-transform infrared spectroscopy) spectra were recorded on KBr pellets using a JASCO FT/IR-4200 apparatus (Shimadzu, Kyoto, Japan) for the “extracted” series and using a Cary 630 FT-IR spectrophotometer (Agilent Technologies LDA, Penang, Malaysia) for the “calcined” series.

Porosity and texture have been measured by low-temperature nitrogen sorption using a Quantachrome Nova 1200e apparatus (Quantachrome Instruments, Boynton Beach, FL, USA). Prior to the analysis, the samples were dried and degassed in a vacuum at room temperature for 6 h. The specific surface area was determined by the Brunauer–Emmett–Teller (BET) method in the relative pressure (P/P_0_) range of 0.05–0.25. The micropore surface area and micropore volume were determined using de Boer’s V-t method in the relative pressure range of 0.15–0.40. Pore size distribution was evaluated with a Density Functional Theory (DFT) equilibrium model in the range of 0.05–1. The pore size was determined also by the Barrett–Joyner–Halenda (BJH) method from adsorption and desorption branches. The total pore volumes were determined using the point closest to 1 for the relative pressure.

The TEM investigations were performed using a Cs-corrected 200 kV Themis (Thermo Fisher, Waltham, MA, USA) electron microscope. Powders were suspended in water, sonicated in a US bath, and dripped on carbon-coated copper grids (Ted Pella 01822-F, Ted Pella Inc., Redding, Canada) with subsequent drying in air at room temperature.

SEM imaging was performed on a LEO 1540 XB (Zeiss, Oberkochen, Germany) instrument at low accelerating voltage (2 kV) and low beam current (40 pA). The powder was dispersed on a double-sided carbon tape to provide better conductivity during the measurement.

Small-angle X-ray scattering measurements were performed with a SAXSPoint 2.0 instrument (Anton Paar Gmbh, Graz, Austria) equipped with an EIGER2 R 1M position-sensitive detector (Dectris, Dättwil AG, Baden, Switzerland). Powdered samples were confined between two sticky tapes on a metallic grid and mounted on a motorized x-y stage. The detector distance was calibrated with silver behenate. The measurements were carried out at room temperature in a vacuum.

The ultra-small-angle neutron scattering (USANS) technique has been used to reveal the overall morphology of the nanoparticles in the size range from 100 nm to 1 µm. The measurements were performed with the USANS instrument MAUD [19] operating at the thermal channel of the LVR15 10 MW research reactor in Řež, Czech Republic. Samples were filled in quartz cuvettes of a 2 mm flight path and measured in air at ambient conditions. A model of spherical particles with log-normal distribution of sizes was used to fit the experimental data. The fitting was performed using the SASprofit software (version 5.0.5.1.) [20].

### 2.3. Metal Ion Sorption Experiments

For the adsorption experiments, a 1000 mg/L Pb(NO_3_)_2_ (Sigma-Aldrich, Schnelldorf, Germany) stock solution was prepared in deionized water, and the pH was adjusted to 5.0 using HCl and NaOH. All other metallic ion solutions were prepared from this solution with appropriate dilution using pH 5 deionized water. A total of 8 mg of sorbent was filled into 50 mL Falcon-type conical plastic tubes and 30 mL of metal solutions were added in the concentration range of 10–500 mg/L. The mixtures were stirred using a Julabo SW22 (Julabo GmbH, Seelbach, Germany) shaking water bath at 25 °C for 4 h to reach equilibrium. The supernatant was separated by centrifugation, and the metal ion concentration in the supernatant was measured using an inductively coupled plasma optical emission spectrometer PerkinElmer Avio 200 (PerkinElmer, Shelton, CT, USA). The measurements were performed in duplicate.

## 3. Results and Discussion

### 3.1. Infrared Spectroscopy

**Figure 2:**
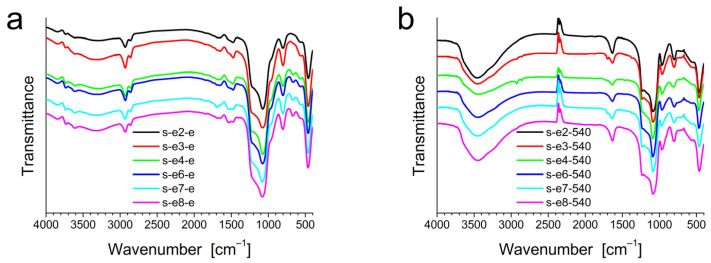
Infrared spectra of the solvent-extracted (**a**) and calcined (**b**) mesoporous silica.

Figure 2 shows the FT-IR spectra acquired from all the synthesized samples. Vibration bands of molecular water, hydrogen-bonded to each other and to SiOH groups, are observed around 3735 cm^−1^, 3610 cm^−1^, and 3465 cm^−1^ [21]. The characteristic vibration bands from the alkyl tails of the surfactant molecules around 2920 cm^−1^ and 2850 cm^−1^, due to the asymmetric and symmetric stretching vibrations, respectively, of the methylene groups [22], were observed in the “extracted” series samples, indicating the incomplete surfactant extraction. C–H bending in methyl/methylene groups is observable at 1477 cm^−1^ [23,24]. All samples show the vibration bands of the silica skeleton around 1050, 800, and 450 cm^−1^, corresponding to the asymmetric stretching, symmetric stretching, and bending vibration of the Si-O-Si network, respectively [21,22,25]. The presence of the silanol groups was confirmed by the band centered at about 960 cm^−1^, associated with the stretching mode of the Si-OH groups [26,27,28]. The band assignments are shown in Table 1.

### 3.2. Nitrogen Sorption Isotherms

**Figure 3:**
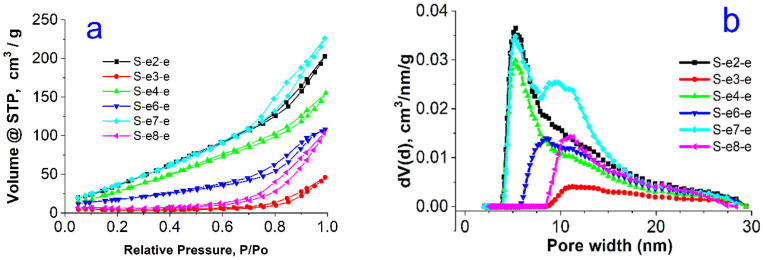
Nitrogen sorption isotherms (**a**) and pore size distributions (**b**) for the solvent-extracted samples.

**Figure 4:**
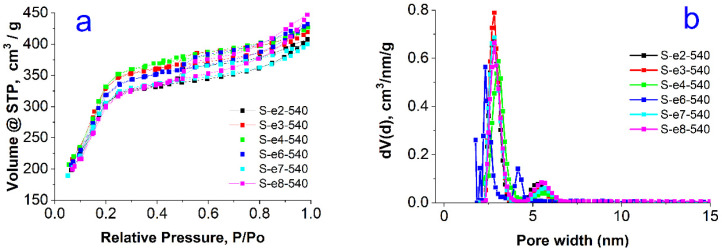
Nitrogen sorption isotherms (**a**) and pore size distributions (**b**) for the calcined series of samples.

The N_2_ adsorption–desorption isotherms are shown in Figure 3a and Figure 4a. All materials present a type IVb isotherm according to the IUPAC classification. Type IVb is specific for mesoporous materials having conical or cylindrical pores, closed at one end [29,30]. The presence of hysteresis indicates that pore condensation takes place. The narrow hysteresis loop closing near 0.2 P/P_0_ suggests the presence of some wide pores that could have more access to the external surface [30]. A H2(b) type hysteresis has been observed for all samples. This type of hysteresis is encountered in samples where pore blocking takes place and pores have the shape of ink bottles with larger necks [29,30].

The pore size distribution (Figure 3b and Figure 4b) is broad and centered around 5 nm for the extracted series, and appears to be bimodal for the calcined samples, with the majority of pore sizes centered around 3.5 nm and a small fraction of pores with diameters around 5 nm.

All textural parameters for surface area, total pore volume, and mean pore diameter are summarized in Table 2. The specific surface area of the calcined samples is very high, around 1200 m^2^/g, whereas these values are substantially lower for the extracted series, not exceeding 169 m^2^/g. The total pore volumes are around 0.65 cm^3^/g for the samples from the calcined series and are much smaller for the samples after solvent extraction. Furthermore, they vary in a broad range non-systematically for this series, indicating the quasi-random degree of the template extraction achieved by the treatment with acidified ethanol.

The different texture of the materials is also observed in the surface fractal dimensions evaluated by the Frenkel–Halsey–Hill (FHH) method [31]. The FHH model allows one to calculate the fractal geometry of the surfaces and determine their irregularities and porous structure. A value of D_f_ = 2 indicates a smooth surface and 3 is characteristic of a rough, porous, or sponge-like surface [31]. The FHH data (Table 2) indicate that using the calcination method for the extraction of the gemini surfactant, a more porous structure is obtained compared with the extraction with the mixture of solvents. This result is related closely to the fact that in the used procedure only a part of the surfactant molecules could be removed from the porous silica particles.

### 3.3. Scanning Electron Microscopy

**Figure 5:**
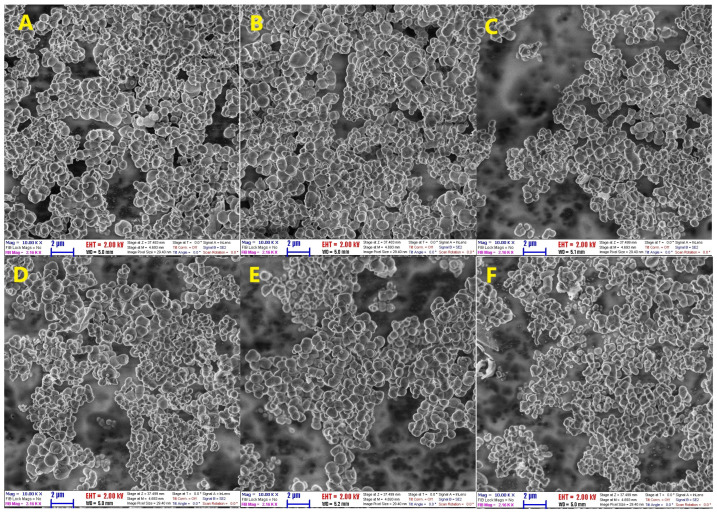

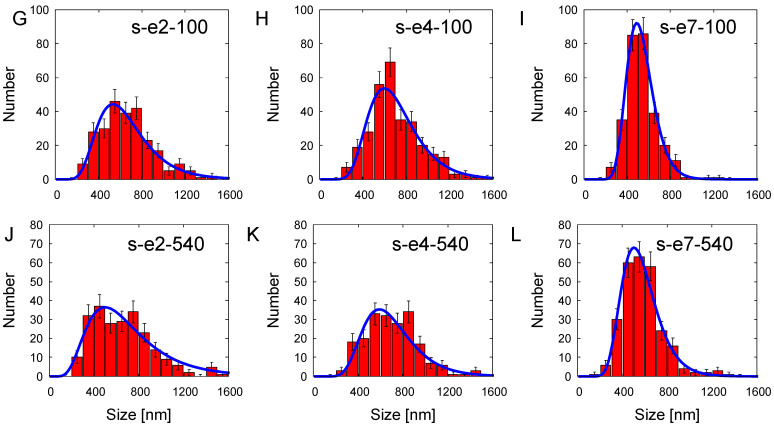
SEM micrographs and particle size distribution histograms for the as-prepared and calcined samples. The solid lines are fits of log-normal size distribution. (**A**,**G**): S-e2-100; (**B**,**H**): S-e4-100; (**C**,**I**): S-e7-100; (**D**,**J**): S-e2-540; (**E**,**K**): S-e4-540 and (**F**,**L**): S-e7-540.

SEM micrographs reveal nearly similar, close to spherical particle morphologies for all samples prepared with different gemini molecules, with unimodal size distribution. Most of the particles are of sizes between 400 and 800 nm, while a non-negligible number of larger particles is present in all samples, with sizes up to 2 micrometers. Micrographs of three representative samples, before and after calcination, are shown in Figure 5, together with the calculated particle size distributions.

To obtain more detailed, quantitative characteristics, the sizes of particles were measured for all samples by selecting randomly at least 250 particles in several images. The size distributions of the particles, calculated by ImageJ, were then fitted to both Gaussian and log-normal functions. Comparing the reduced chi-square values for all samples, it was confirmed that the quality of fit for the log-normal distribution is better than that for the Gaussian distribution. By contrasting the size distribution functions for the same samples (S-e2, S-e4, and S-e7) dried at different temperatures of 100 °C and 540 °C, it was seen that the mean sizes of particles overlap within the combined error bars, indicating that the particle size did not change after high-temperature calcination (Table 3). Another notable observation is that the size distributions corresponding to the samples dried at high temperatures are broader than those of the samples dried at low temperatures. The largest mean particle size is observed for sample S-e4, prepared with gemini molecules having four carbons in the spacer, and the size decreases slightly with the addition of more methylene groups to the spacer chain.

### 3.4. Transmission Electron Microscopy

**Figure 6:**
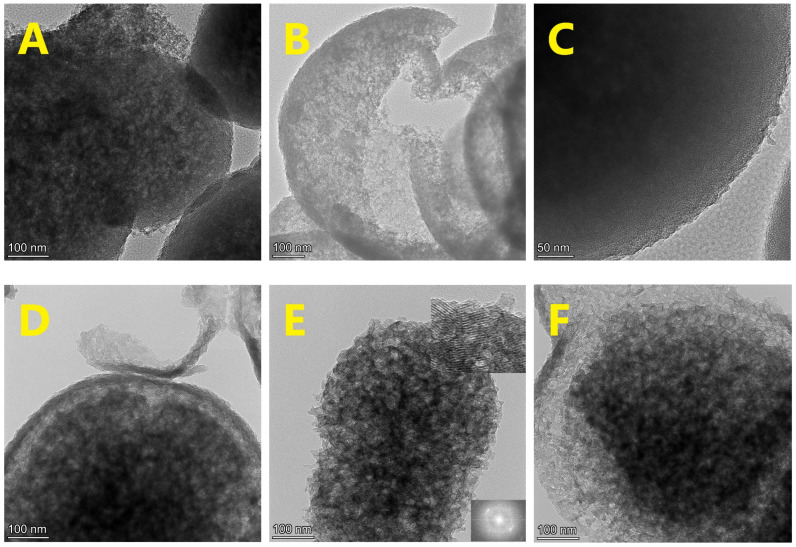

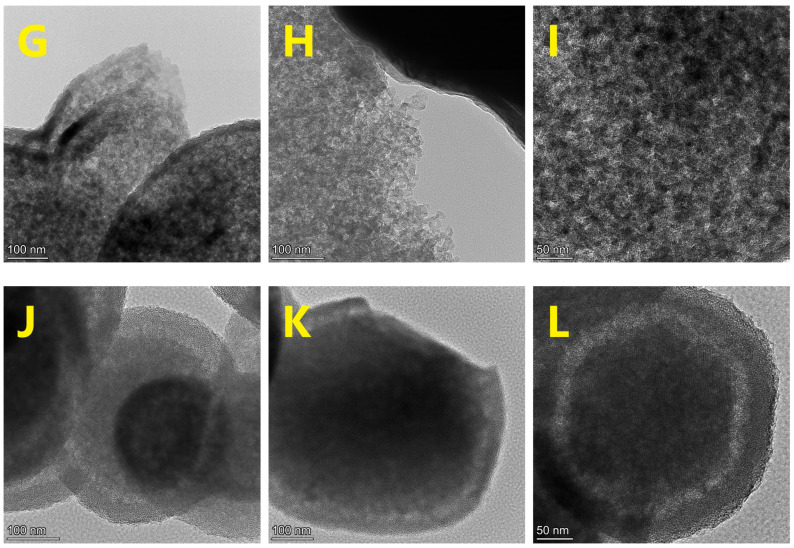
TEM overview images of mesoporous silica prepared with gemini surfactants of different spacer lengths, obtained with magnifications appr. 70 k and 120 k. (**A**–**C**): S-e2-540; (**D**–**F**): S-e3-540; (**G**–**I**): S-e6-540; (**J**–**L**): S-e8-540. The inset in the upper right corner of panel “E” is a magnified part of the same particle. The inset in the lower right corner of panel “E” is a Fast Fourier pattern of the image indicating the regular arrangement of the pores.

TEM analysis was conducted on four samples selected to represent the materials prepared using the gemini surfactants with short, medium, and long spacer lengths. Most of the images revealed particles in the size range of 0.5–1 µm. Selected samples are shown in Figure 6. The samples appear to have spherical or slightly elongated morphologies, and in some cases, broken or fractured particles can be seen (Figure 6B,D,G). Some particles have a darker core surrounded by a two-layer shell (e.g., Figure 6B,D), and can be considered as partially hollow spherical particles, showing similarity with the smaller and more regular hollow spherical particles reported by Li et al. [15].

At higher magnification, most of the particles are seen to be built of ~15 nm-sized domains, each consisting of wide parallel channels (Figure 6E, inset in upper corner). Fringes of the porous channels can be observed with a periodicity of ~2.85 nm, as obtained by the FFT (Figure 6E, inset in lower corner). Notably, also the second harmonic can be observed, showing a long-range periodicity. The orientations of the ~15 nm domains are random, indicating the complex formation of the micron-sized silica particles from smaller primary particles, which had already formed around a liquid crystalline micellar domain.

### 3.5. Small-Angle X-Ray Scattering

**Figure 7:**
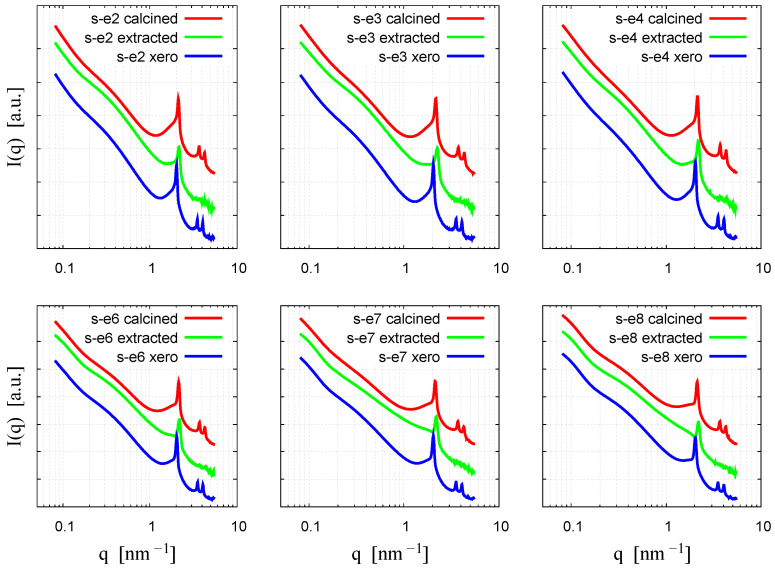
SAXS diffractograms of mesoporous silica materials prepared with gemini surfactants of different spacer lengths. In all graphs the lower curve corresponds to the “xero” samples, the middle curve to the solvent-extracted curves, and the upper curve to the calcined curves.

The small-angle diffraction patterns for the three series of xerogels, calcined materials, and solvent-extracted materials are shown in Figure 7. All samples show two characteristic features: a steep power-law-like decay in the small q range, and a series of sharp diffraction peaks in the high q range. The power-law decay is due to the interface scattering of large silica particles, common to the materials obtained in the Stöber process [32]. The broad wave in the q range 0.2–1 nm^−1^ reflects the ~10–50 nm size inhomogeneity in the particle structure, which can correspond to the ~15 nm sized domains observed in the TEM.

**Figure 8:**
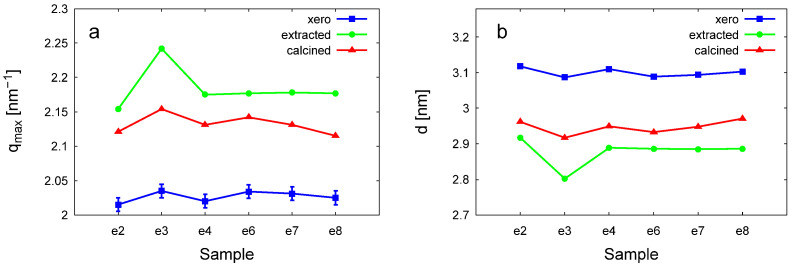
Position of the (10) reflection (**a**) and the corresponding lattice spacings (**b**) for the three series of gemini surfactant-templated silica materials.

The three sharp diffraction peaks at q values proportional to 1:√3:2 can be indexed as the reflections corresponding to the (10), (11), and (20) lattice planes of the two-dimensional hexagonal lattice. These peaks reveal a well-organized crystalline structure, which can be identified as that of MCM-41 materials [31,32,33,34,35]. The peak positions and the corresponding lattice spacings are shown comparatively in Figure 8 and listed in Table 3.

For the xerogel samples, the peak positions were similar for the materials prepared with the gemini surfactants of different spacer lengths (Table 3). The first diffraction peak appeared at q_(01)_ = 2.03 ± 0.005 nm^−1^, which corresponds to lattice spacing of 3.11 nm. This is the expected behavior for surfactant-templated ordered porous silica, as the pore size and the lattice parameter are determined by the surfactant alkyl tail, which is similar for all six compounds. Calcination and the treatment with acidified ethanol caused a shrinkage of the hexagonal structure, similar to the behavior of MCM-41 prepared with an alkyl-trimethylammonium micellar template [33,34,35,36,37,38]. Interestingly, this shrinkage was markedly different among the materials prepared with the gemini surfactants with different spacer lengths. The degree of shrinkage shows the structural strength of the silica framework and was the highest for the samples S-e3-e and S-e3-540, prepared with a relatively short spacer surfactant (Figure 8b). During calcination, the micellar template was fully eliminated, according to the IR spectra and the BET-specific surface, whereas solvent removal was not as efficient, leaving a fraction of the surfactants in the material. Furthermore, the solvent removal partly destroyed the hexagonal pore structure, as seen by the disappearance of the higher-order reflections.

The removal of the surfactant also led to the broadening of the diffraction peaks, showing a decrease in the long-range order. The apparent size of the crystalline domains can be calculated from the width of the second reflection using the Scherrer equation. Comparing the same samples dried at different temperatures (540 °C and 100 °C), the diffraction peaks broaden, and the domain size decreases as the drying temperature increases (Table 3). These results indicate that the commonly used procedure of calcination at 540 °C leads to a decrease in crystalline order. Another interesting observation is that increasing the number of carbon atoms in the spacer of the gemini molecule results in a decrease in the rate of reduction in the domain size. For example, the domain size of the S-e2-540 sample decreased by 39% after heating, while for S-e8-540, it decreased by only 19% (Table 3). This can be attributed to the different strengths of the silica network in response to the different morphologies of the micelles forming the pore template in the gelation process.

### 3.6. Ultra-Small-Angle Neutron Scattering

**Figure 9:**
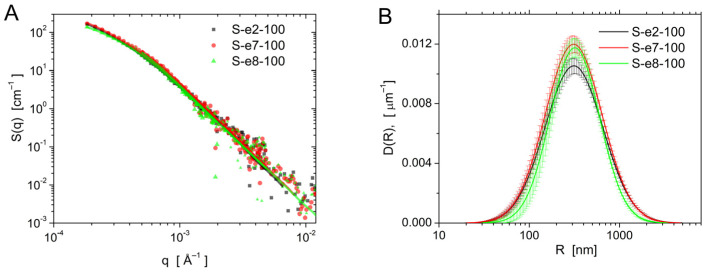
USANS data for samples S-e2-100, S-e7-100, and S-e8-100 and fits of the form factor of spherical particles with log-normal size distribution (**A**). The obtained size distributions (**B**).

The USANS measurements allow us to assess the overall dimensions and morphology of the nanoparticles. The scattering intensity data are shown in Figure 9A, together with the fitted model curves. It can be seen that the data for the three samples overlap, showing that the mean particle sizes are very similar in each sample. Similarly to the analysis of the particle sizes by SEM, a theoretical scattering model of log-normal size distribution, smeared by the instrumental resolution, was applied to the data (Figure 9A). The size distribution of the assumed spherical particles is shown in Figure 9B and the parameters obtained by data fitting are collected in Table 3. While the calculated mean sizes are very close to each other, a weak trend can be noticed, according to which the increase in the spacer length in the template gemini molecule leads to the development of particles of smaller sizes. The mean particle size obtained in the USANS measurements was consistently larger than the SEM observations. This difference can be attributed to the structure factor of the agglomerated particles, which is generally unknown for a loosely agglomerated particle system and was not considered in the modeling.

### 3.7. Adsorption Isotherm of Pb(II)

**Figure 10:**
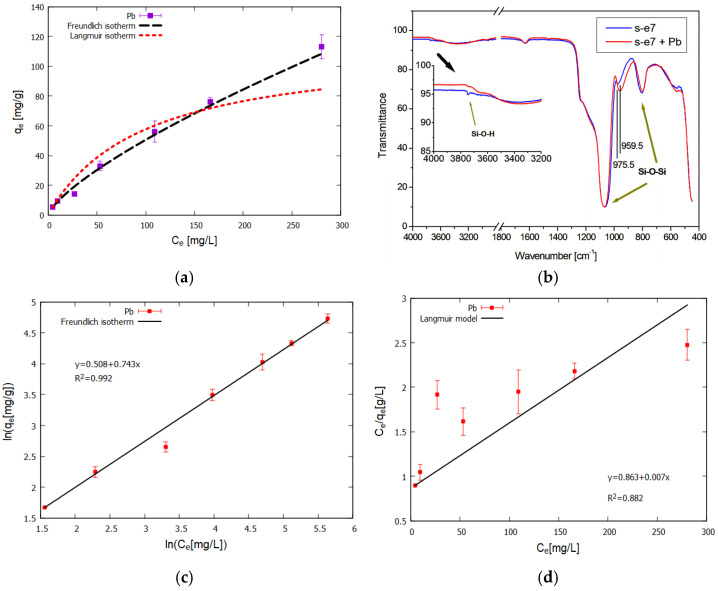
Adsorption isotherms of sample S-e7-540 for Pb(II) at T = 25 °C. (**a**) Experimental data and non-linear least square fit curves to Langmuir and Freundlich model equations; (**b**) FTIR spectra of the sample before and after sorption; (**c**,**d**) linear representation and linear fits to Freundlich and Langmuir isotherm equations.

Metal ion adsorption experiments were conducted for the calcined sample S-e7-540 to investigate the adsorption performance of the novel mesoporous materials. The adsorption isotherms were analyzed with the two commonly used Langmuir and Freundlich models [39,40]. We focused on the determination of the main characteristics of sorption, which can be classified as predominantly monolayer, Langmuir-type behavior, or heterogeneous sorption, where the sorption sites have different binding energies, and which can occur in the case of multilayer coverage of the sorbent surfaces. The heterogeneous sorption can conveniently be described with the empirical Freundlich model. We did not test further popular three-parameter isotherm models, given the finite accuracy of the experimental data points, and because the observed sorption behavior aligns well with the Freundlich model, as seen by visual comparison of the graphs of the theoretical fitted models and the data (Figure 10), as well as by the R^2^ values, which show the superior fit to the Freundlich isotherm. The experimental isotherm shape indicates that a plateau stage is not reached, suggesting that the materials’ adsorption sites are not saturated at the given conditions, and further adsorption of Pb(II) is possible at higher initial ion concentrations. The experimentally determined sorption capacity for the S-e7-540 material was 110 mg Pb(II)/g (sorbent), with a 10% experimental uncertainty (Figure 10a). In Table 4 the fitted parameters for the Langmuir and Freundlich isotherms are collected. IR spectra of the sample before and after the adsorption are shown in Figure 10b and reveal the change in the surface silanol vibrations due to the partial replacement on the binding sites of H^+^ by Pb^2+^ cations: the O–H stretching vibration of the silanol group at 3742 cm^−1^ becomes much weaker, and the Si–OH stretching mode blue-shifts from 975.5 to 959.5 cm^−1^.

The adsorption capacity of these materials was compared to some other adsorbents from the recent literature (Table 5). Concerning the Pb(II) adsorption, the obtained value of 113.85 mg/g proved that the mesoporous silica prepared with the gemini surfactant as a template showed higher adsorption properties compared with many other mesoporous silica sorbents. The reason for the revealed high performance can be, beyond the high internal surface, the partially hollow and granular structure of the nanoparticles, allowing for higher penetration of the solvent and the sorbate into the interior of the particles. This morphology is most likely the consequence of the specific self-assembly behavior of the gemini surfactants, which differs from the typical cylindrical micelle shape of the single alkyl chain CTAB surfactant, commonly used in the synthesis of MCM-41 materials.

## 4. Conclusions

Mesoporous silica materials were prepared using sol–gel technology on a supramolecular liquid crystal template consisting of gemini surfactant micelles in alkaline conditions. Their structure has been analyzed by a variety of physicochemical methods, including FT-IR spectroscopy, nitrogen porosimetry, SAXS, SANS, and scanning and transmission electron microscopy. The diester gemini surfactants with both short and long polymethylene spacer chains produced highly ordered mesoporous materials with high specific surfaces, evidenced by the sharp X-ray reflections and the nitrogen porosimetry data. The materials consisted of submicron-sized, close to spherical particles of the MCM-41 type, according to the electron microscopy data and the characteristic low-angle diffraction pattern. TEM images show domains with hexagonal ordering of pores and a characteristic hollow sphere morphology. A large part of the particles is fragmented into domains of sizes of about 20 nm. SAXS data show that the extraction of the gemini surfactant from the pores by high-temperature calcination caused a contraction of the 2-dimensional hexagonal lattice by a factor of ca. 0.6–0.8. Surfactant removal by acidic ethanol resulted in a somewhat larger shrinkage and a partial destruction of the hexagonal order. The materials were tested for adsorption of Pb(II) in a batch sorption experiment and showed an adsorption capacity of 113 mg/g, which is higher than that of most silica-based sorbents reported in the recent literature. This sorption capacity can be attributed to the hierarchical fractured structure of the mesoporous silica nanoparticles.

## Figures and Tables

**Table 1:** The assignment for the main FT-IR bands observed in the two series of gemini-functionalized mesoporous silica.

Band Assignments	S-e_x_-e (Solvent-Extracted) ν [cm^−1^]	S-e_x_-540 (Calcined) ν [cm^−1^]
Free silanol groups and molecular water [21]	3735, 3610	3465
Methylene asymmetric stretching [22]	2932	
Methylene symmetric stretching [22]	2861	
Molecular water and the SiO_2_ network [24]	1650	1624
Methylene C–H bending [23,24]	1477	
Asymmetric Si–O–Si stretching [25]	1075	1086
Si–OH stretching [26,27,28]		957
Symmetric Si–O–Si stretching [25,26]	809	791
Si–O–Si bending [21,25]	460	460

**Table 2:** Textural parameters of the mesoporous silica samples obtained by nitrogen adsorption.

Sample	Surface Area, m^2^/g	Micropore Surface Area, m^2^/g	BJH ads, nm	BJH des, nm	DFT Pore Size, nm	Total Pore Volume, cm^3^/g	Micropore Volume, cm^3^/g	FHH (ads) D_f_
S-e2-e	169		3.43	3.17	5.28	0.32		1.67
S-e3-e	11		12.52	3.33	11.28	0.07		1.42
S-e4-e	137		3.43	3.09	5.29	0.24		1.75
S-e6-e	67		3.43	3.97	8.46	0.17		1.68
S-e7-e	169		3.47	3.34	5.29	0.35		1.58
S-e8-e	11		6.66	7.59	10.89	0.16		0.87
S-e2-540	1214	706	3.04	3.96	2.82	0.63	0.24	2.67
S-e3-540	1331	779	3.39	3.95	2.82	0.65	0.26	2.70
S-e4-540	1309	711	3.10	3.40	3.06	0.66	0.24	2.69
S-e6-540	1268	708	3.06	3.37	2.31	0.67	0.24	2.66
S-e7-540	1195	683	3.46	3.96	2.82	0.62	0.24	2.68
S-e8-540	1201	621	3.05	3.96	2.82	0.69	0.20	2.61

**Table 3:** Structural parameters of the mesoporous silica samples obtained by SEM, SAXS, and USANS.

Sample	(10) Peak Position [nm^−1^]	d [nm]	Domain Size [nm]	Reduction in Domain Size upon Calcination	Diameter and HWHM by SEM [nm]	Diameter and HWHM by USANS [nm]
S-e2-100	2.02	3.11	45.2		615.9, 270	713, 31
S-e2-540	2.12	2.97	30.1	0.61	604.5, 321	
S-e3-100	2.03	3.09	41.0		555.3, 210	
S-e3-540	2.14	2.93	26.5	0.62		
S-e4-100	2.02	3.11	42.4		669.1, 255	
S-e4-540	2.12	2.96	32.0	0.73	655.9, 248	
S-e6-100	2.03	3.09	40.1		569.3, 233	
S-e6-540	2.13	2.95	30.2	0.73		
S-e7-100	2.03	3.10	40.3		513.8, 135	685, 21
S-e7-540	2.12	2.96	29.6	0.72	547.5, 173	
S-e8-100	2.03	3.09	36.7		530.7, 188	581, 20
S-e8-540	2.13	2.95	31.2	0.82		

**Table 4:** Fitting parameters of Langmuir and Freundlich adsorption isotherms of Pb(II) on the gemini-surfactant templated mesoporous silica at pH = 5.0 and T = 25 °C.

Langmuir Isotherm			Freundlich Isotherm		
K_L_ [L mg^−1^]	Q_m_ [mg g^−1^]	R^2^	*n*	K_F_ [mg g^−1^]	R^2^
0.0102	113.852	0.882	1.3512	1.671	0.992

**Table 5:** Comparison of Pb(II) adsorption capacity of gemini-surfactant templated mesoporous silica with some other adsorbents based on ordered mesoporous silica.

Adsorbent	Synthesis Method	Pb(II) Adsorption Capacity [mg/g]	Reference
MCM-41	MCM-41 from TEOS and diester gemini surfactant in alkaline media; template removal by calcination	113.85	The present work
MCM-41	MCM-41 from TEOS and CTAB in alkaline media; template removal by calcination	18.8	[41]
NH_2_-MCM-41	MCM-41 prepared from TEOS, APTES, and CTAB in alkaline media by co-condensation; template removal by solvent extraction	64.2	[42]
NH_2_-MCM-41	MCM-41 prepared from TEOS and CTAB in alkaline media; post-synthesis functionalization with APTES	57.7	[43]
NH_2_-MCM-48	MCM-48 prepared from TEOS and CTAB in alkaline media; post-synthesis functionalization with APTES	75.2	[44]
SH-MCM-48	MCM-48 prepared from TEOS and CTAB in alkaline media; post-synthesis functionalization with MPTMS	31.2	[44]
Chitosan-MCM-41	MCM-41 prepared from Na silicate and CTAB; subsequent grafting with chitosan	90.91	[45]
Bifunctionalized NH_2_-SH-MCM-41	MCM-41 prepared from TEOS and CTAB in alkaline media; subsequent post-grafting by hydrolyzed APTES and MPTMS	55.56	[46]
SH-MCM-41	MCM-41 prepared from TEOS, MPTMS, and CTAB in alkaline media by co-condensation; template removal by solvent extraction	66.04	[47]

## Data Availability

The original contributions presented in this study are included in the article. Further inquiries can be directed to the corresponding authors.
